# Supplementary material for: EnzML: multi-label prediction of enzyme classes using InterPro signatures
Source: BMC Bioinformatics. 2012 Apr 25;13:61. doi: 10.1186/1471-2105-13-61 (PMC3483700; doi:10.1186/1471-2105-13-61)
Supplement: Addtional file 5 — The Java code to format the data files, evaluate and predict. The file enzml_java_code.tar.gz contains the Java code used to format database data to ARFF and XML formats, to execute cross and train-test (jackknife) evaluations and to record evaluation results to database. More information is included in the readme.txt file and the Javadoc files. The code can be used with a MySQL database. To use a different database software, other JDBC drivers might be required. [file 1471-2105-13-61-S5.gz › java_code/utils/doc/uk/ac/ed/inf/utils/guiutils/SimpleRadioButtonPanel.html]

SimpleRadioButtonPanel


---


|  |  |  |
| --- | --- | --- |
| **Overview** \| **Package** \| **Class** \| **Use** \| **Tree** \| **Deprecated** \| **Index** \| **Help** | |  |
| **PREV CLASS**   NEXT CLASS | **FRAMES**    **NO FRAMES**     **All Classes** |  |
| SUMMARY: NESTED \| FIELD \| CONSTR \| METHOD | DETAIL: FIELD \| CONSTR \| METHOD |  |


---


## uk.ac.ed.inf.utils.guiutils Class SimpleRadioButtonPanel

```
java.lang.Object
  uk.ac.ed.inf.utils.guiutils.SimpleRadioButtonPanel
```

---

``` public class SimpleRadioButtonPanel extends java.lang.Object ```

---

| **Field Summary** | |
| --- | --- |
| `java.lang.Object` | `choiceEvent` |


**Constructor Summary**

`SimpleRadioButtonPanel(java.lang.String[] radioOptions,
java.lang.String[] comments)`
  
          Generates a simple radio button popup


| **Method Summary** | |
| --- | --- |
| `java.lang.String` | `getChosenOption()` |
| `static void` | `main(java.lang.String[] args)`             For testing |
| `void` | `setChosenOption(java.lang.String option)` |

**Methods inherited from class java.lang.Object**

`equals, getClass, hashCode, notify, notifyAll, toString, wait, wait, wait`

**Field Detail**

### choiceEvent

```
public java.lang.Object choiceEvent
```


**Constructor Detail**

### SimpleRadioButtonPanel

```
public SimpleRadioButtonPanel(java.lang.String[] radioOptions,
                              java.lang.String[] comments)
```

:   Generates a simple radio button popup

    **Parameters:**: `radioOptions` - the options for each radio button: `comments` - the text/comments beside each radio button


**Method Detail**

### getChosenOption

```
public java.lang.String getChosenOption()
```

---


### setChosenOption

```
public void setChosenOption(java.lang.String option)
```

---


### main

```
public static void main(java.lang.String[] args)
```

:   For testing

    :   **Parameters:**: `args` -


---


|  |  |  |
| --- | --- | --- |
| **Overview** \| **Package** \| **Class** \| **Use** \| **Tree** \| **Deprecated** \| **Index** \| **Help** | |  |
| **PREV CLASS**   NEXT CLASS | **FRAMES**    **NO FRAMES**     **All Classes** |  |
| SUMMARY: NESTED \| FIELD \| CONSTR \| METHOD | DETAIL: FIELD \| CONSTR \| METHOD |  |


---
